# Oral allergy syndrome

**DOI:** 10.1186/1939-4551-8-S1-A191

**Published:** 2015-04-08

**Authors:** Luciana Canela

**Affiliations:** 1 Hospital Central Da Policia Militar, Brazil

## Background

The purpose of this study is to describe an adverse reaction to eating lettuce.

## Methods

Case report.

## Results

L.V.C.L., female, 12 years old, from Rio de Janeiro, Brazil, was attended in march 2012 at the clinic of Allergy and Immunology, with moderate persistent rhinitis. She had a positive skin test to *Dermatophagoides pteronyssinus* and specific immunotherapy was started. In September 2012, patient reported severe itching in the oral cavity and pharynx when eating lettuce. She denied symptoms similar to any other food. In dezembro 2012, was performe "prick to prick" for lettuce, with the following result: histamine=7mm; negative control=0mm and lettuce=9 mm. Patient was instructed not to eat lettuce. The test was confirmed on another occasion and was also performed in the patient's grandmother, who did not have a history of reaction to lettuce, with the same lettuce tested in the patient, to exclude the presence of irritants as a cause of positive skin test. The result of patient's grandmother was: histamine=5 mm; negative control=0mm and lettuce=0mm. In May 2013, was performed RAST (radioallergosorbent) test for lettuce and result was negative. The patient was advised to maintain an exclusion diet of lettuce but she decided to eat lettuce on their own. By eating organic and non-organic lettuce, the patient presented severe oropharyngeal pruritus, as reported in future consultations.

## Conclusions

Oral allergy syndrome is very frequent and is associated with intake of various foods, especially vegetables and fruits. Adverse reactions to lettuce are rare, with few cases described throughout the world. The "prick to prick" has a higher accuracy for the diagnosis of allergic conditions, exceeding the RAST test. Intake of lettuce and onset of symptoms confirms the diagnosis. It is essential to enhance the clinical history to make the diagnosis of food allergy, even for food less likely.

## Consent

Written informed consent was obtained from the patient for publication of this abstract and any accompanying images. A copy of the written consent is available for review by the Editor of this journal.

